# COVID-19 and Cancer: Lessons Learnt from a Michigan Hotspot

**DOI:** 10.3390/cancers12092377

**Published:** 2020-08-22

**Authors:** Sunny R. K. Singh, Kannan Thanikachalam, Hiba Jabbour-Aida, Laila M. Poisson, Gazala Khan

**Affiliations:** 1 Division of Hematology and Medical Oncology, Henry Ford Health System, Detroit, MI 48202, USA; kannanmbbs@gmail.com (K.T.); hjabbou1@hfhs.org (H.J.-A.); gkhan1@hfhs.org (G.K.); 2 Department of Public Health Sciences, Henry Ford Hospital, Detroit, MI 48202, USA; lpoisso1@hfhs.org

**Keywords:** COVID-19, Sars-CoV-2, cancer, health care disparity

## Abstract

(1) Background: Outcomes with coronavirus disease 2019 (COVID-19) have been worse in those with comorbidities and amongst minorities. In our study, we describe outcomes amongst cancer patients in Detroit, a major COVID-19 hotspot with a predominant inner-city population. (2) Methods: We retrospectively analyzed 85 patients with active invasive cancers who were infected with COVID-19. The primary outcome was death or transition to hospice. (3) Results: The majority were males (55.3%, n = 47), ≤70 years old (58.5%, n = 50), and African Americans (65.5%, n = 55). The most common primary site was prostate (18.8%, n = 16). Inpatient admission was documented in 85.5% (n = 73), ICU admission in 35.3% (n = 30), and primary outcome in 43.8% (n = 32) of hospitalized patients. On a multivariate analysis, factors associated with increased odds of a primary outcome included an age of >70 years versus ≤70 years (OR 4.7, *p* = 0.012) and of male gender (OR 4.8, *p* = 0.008). Recent cancer-directed therapy was administered in 66.7% (n = 20) of ICU admissions versus 39.5% (n = 17) of general floor admissions (Chi-square *p*-value of 0.023). (4) Conclusions: High rates of mortality/transition to hospice and ICU utilization were noted amongst our patients with active invasive cancer, following a COVID-19 infection. Men and those of >70 years of age had a greater than four-fold increase in odds of death or transition to hospice.

## 1. Introduction

As of 13 May 2020, a total of 48,391 cases of coronavirus disease 2019 (COVID-19) were identified in Michigan, with deaths amounting to 4714 (case fatality rate of 10%) [1]. COVID-19 is typically more severe and lethal amongst the elderly and those with medical comorbidities [2,3,4,5]. Cancer was found to contribute towards adverse clinical outcomes in those with COVID-19 infection [6]. Much of the early data of COVID-19 in the cancer patient population was derived from patients treated in Wuhan, China. The first Chinese nationwide analysis was a prospective cohort study that included 18 patients with cancer and COVID-19 [7]. They reported a higher risk of severe events (defined as death or being admitted to the intensive care unit requiring invasive ventilation) in those with cancer compared to those without cancer. In the study by Zhang and colleagues, the authors looked at 28 patients with solid tumors among 1276 COVID-19 patients admitted to three hospitals in Wuhan, China [8]. The most common primary site was lung and the mortality rate (28.6%) was more than ten times higher than that reported in all COVID-19 patients in China [9]. In addition, they identified that the recent use of anticancer therapies within 14 days of infection was an independent predictor of death or other severe events. Recent data from the US have been corroborative. In a study from New York, amongst 334 cancer patients infected with COVID-19, the most common primary site was breast (n = 57) or prostate cancer (n = 56) and patients under the age of 50 with cancer had a five-fold higher mortality than those in the same age group without cancer [10]. In the study published by COVID-19 and Cancer consortium, mortality was reported at 13%. However, about 45% patients were in remission or had no evidence of disease and 11% had an unknown/missing status of the cancer. Only 16% of the patient population in this study were non-Hispanic black and 13% of those with progressing/stable/responding cancers were admitted to the intensive care unit. Of the 396 patients with progressing/stable/responding (active cancer), 16.6% patients died and 13.1% were admitted to the ICU [11]. Outcomes in our patient population were much worse as shown below. In addition to this, in a recently published larger study from our institution, amongst the 2541 patients included in the analysis, 18.1% died, 24.2% were admitted to the ICU, and 15% (n = 308) had cancer. Of note, the authors did not distinguish between active or past/resolved cancer, nor did they report mortality specifically for cancer patients. Even though cancer was not a factor associated with mortality on multivariate analysis, this should be interpreted with caution. To put into perspective, in our study from the same institution, amongst the 301 patients with COVID-19 and cancer, only 85 (28%) had an active invasive disease. It is possible that the impact of active cancer on mortality was diluted by counting it together with the past/resolved cases, thus yielding a non-significant *p*-value in this study [12]. Despite this limitation, it does give us a benchmark in terms of COVID-19 mortality for hospitalized patients in the Henry Ford Hospital. 

While these studies give us valuable insight into the outcomes of cancer patients with COVID-19 infection, they have some limitations. An extrapolation of data from China to our patient population is not possible due to differences in demographics and cancer epidemiology. The majority of the published retrospective studies did not clearly distinguish patients with a “history of cancer” from those with “active cancer”. In one of the studies based on 218 cancer patients, active cancer was defined as cancer <1 year, and they had 92 such patients [13]. Moreover, factors pertinent to cancer care, such as change in code status or need of home oxygen at discharge have not been adequately described in the any of the previously published studies.

The Henry Ford Health System is a major health care system providing tertiary level care in Metro Detroit and the surrounding areas. A significant proportion of our patients come from the inner-city, which allows us to assess the impact of socio-economic disparities. In our study, we have assessed the impact of COVID-19 infection specifically in patients with active invasive cancer. Moreover, we have presented a descriptive analysis of those who recently received cancer-directed treatment prior to being diagnosed with a COVID-19 infection. Our study highlights the poor outcomes of COVID-19 infection in our cancer patients and the higher likelihood of having received cancer-directed treatment in those needing ICU care. For the above-mentioned reasons, we think our study is unique and imparts valuable lessons learnt while caring for our cancer patients infected with COVID-19.

## 2. Results

### 2.1. Baseline Characteristics

A total of 85 patients were included in the study and the mean age was 68.9 years (range: 31–90 years). Table 1 describes the baseline characteristics for the entire study population. A Charlson Comorbidity Index (CCI) score was available for 89.4% (n = 76) of the patients. The median CCI score for these 76 patients was 6 (range: 1–16). Of all the subjects, 85.5% (n = 73) had COVID-19-related hospitalization, and the remaining (n = 12) were managed as outpatient. The majority of the patients were admitted to the general floor (50.6%, n = 43), while 35.3% (n = 30) were admitted to the ICU. All patients who were managed as outpatient were followed for occurrence of death/transition to hospice until the end of the follow-up period. The mean duration of their follow-up was 29.7 days (median: 31 days, SD: 5.3, range: 20–36 days) from the time of diagnosis with COVID-19. Table 2 summarizes the results of the lab parameters pertinent to the care of a COVID-19 infection for the entire study population. For those managed as outpatient, these lab parameters were collected around the time of being diagnosed with COVID-19 infection. For those hospitalized, these lab parameters were collected during the period of inpatient stay.

**Figure 1:**
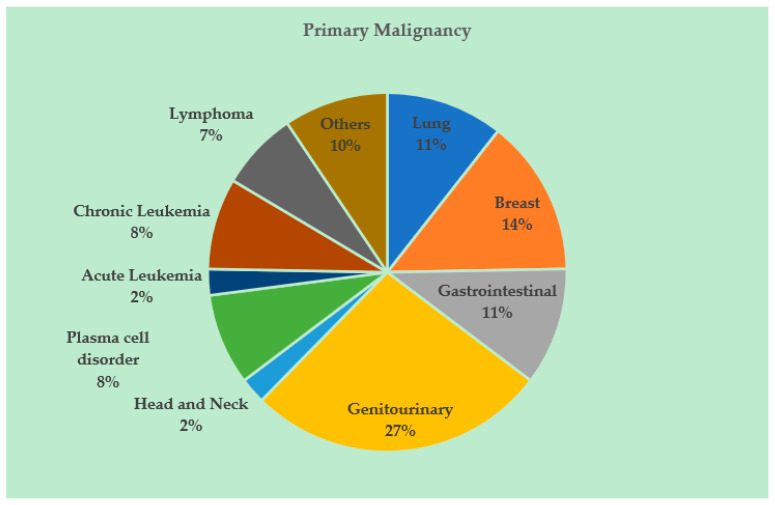
Distribution of the type of primary malignancy in the study population, n = 85 [Other cancers included cutaneous squamous cell cancers (n = 2), cutaneous melanoma (n = 1), sebaceous cell carcinoma (n = 1), leiomyosarcoma (n = 1), bone sarcoma (n = 1), glioblastoma multiforme (n = 1), and cancer of unknown primary (n = 1)].

**Figure 2:**
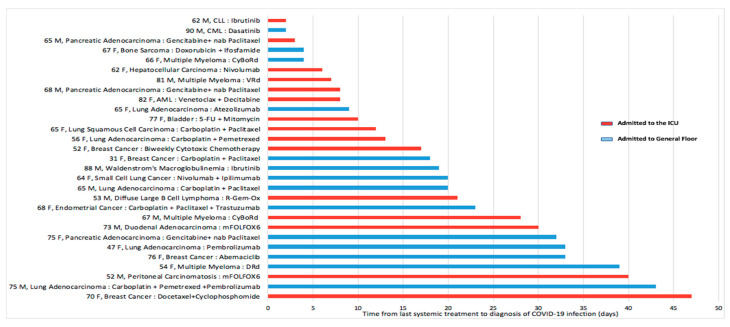
Non-hormonal systemic therapy, time to coronavirus disease 2019 (COVID-19) infection, and type of hospitalization in 29 hospitalized patients. Abbreviations used in this figure were list in Table S1.

Figure 1 shows the breakdown of the entire study population by the type of primary malignancy. The most common primary site was genitourinary (n = 23) followed by breast (n = 12). Amongst the genitourinary cancers, the most common primary site was prostate (69.6%, n = 16). Recent cancer-directed treatment within three months of a COVID-19 infection was documented in 55.3% (n = 47) of patients. The mean time from the latest cancer-directed treatment to COVID-19 infection was 19.1 days (median: 14 days, SD: 18.7, range: 0–83 days). A total of 35 patients received non-hormonal systemic agents, and of these, 82.8% (n = 29) eventually had COVID-19-related hospitalization. More than half of these admissions (51.7%; n = 15) were to the ICU. Figure 2 shows the specific non-hormonal systemic agents administered to these 29 patients (Figure 2). Of these 29 patients, 13 received non-hormonal systemic treatment within 15 days of COVID-19 infection, 9 (69.2%) of which were admitted to the ICU. Of the remaining 16 patients who received non-hormonal systemic treatment beyond 15 days preceding the diagnosis of COVID-19 infection, 6 (37.5%) were admitted to the ICU. Despite not being a statistically significant difference (69.2% vs 37.5%, Chi-square *p*-value of 0.089), this observation is thought-provoking and warrants further investigation.

The median household income for the study population (by zip code of residence) ranged from $21,013 to $123,365. We divided the patients into two groups using a cutoff based on the median household income for the entire state of Michigan ($54,938). Of all the subjects included in the study, 63.3% (n = 50) resided in areas with a median household income of less than $54,938.

#### 2.1.1. COVID-19-Related Hospitalization

Of the 73 patients who were hospitalized, 41.1% (n = 30) were admitted to the ICU and the remaining patients were managed on general floors. The code status was changed to do-not-resuscitate or do-not-intubate or comfort care (DNR/DNI/CC) for 53.4% (n = 39) of the patients. A new deep vein thrombosis or pulmonary embolism (DVT/PE) was documented in only five patients. Of the 73 hospitalized patients, 52.05% (n = 38) were eventually discharged of which 23.7% (n = 9) were discharged on home oxygen. Three patients continued to receive inpatient care until the end of the follow-up period. 

#### 2.1.2. COVID-19-Directed Treatment

Of the entire study population, 77.6% (n = 66) received pharmacological treatments for COVID-19 infection, based on the severity of disease, according to our institutional guidelines. This included hydroxychloroquine in 72.9% (n = 62) of all the patients, steroids in 60% (n = 51), azithromycin in 24.7% (n = 21), and tocilizumab in 3.5% (n = 3). None of the patients managed as outpatient received any of the above. Furthermore, none of the patients received remdesivir.

### 2.2. Primary Outcome (Death or Transition to Hospice) Amongst Hospitalized Patients 

This was noted only amongst the hospitalized patients. None of the patients managed as outpatient died or transitioned to hospice. Of the 73 patients hospitalized in our cohort of active cancer patients for COVID-19 infection, 43.8% (n = 32) died in the same admission or were transitioned to hospice care. Table 1 shows a comparison of baseline characteristics of the hospitalized patients who met the primary outcome (n = 32) versus those who did not (n = 41). A statistically higher proportion of those who met the primary outcome were males, >70 years of age, and those admitted to ICU. Primary outcome was documented in 55.5% (n = 10) of patients with hematological malignancy, 46.1% (n = 12) with metastatic solid cancer, and 34.5% (n = 10) with non-metastatic solid cancer. However, no statistically significant difference was noted amongst the groups (Chi-square *p*-value of 0.351). Table 2 compares the values of the COVID-19 pertinent laboratory parameters measured during the hospitalization for those who died/transitioned to hospice (n = 32) versus those who did not (n = 41). Higher levels of ANC, serum Cr, AST, LDH, Troponin, BNP, CRP, and D-Dimer were noted in those who met the primary outcome versus those who did not, and this difference was statistically significant. Patients with known end stage renal disease/chronic kidney disease and heart failure were excluded when we compared the Cr and Troponin/BNP, respectively, between the two groups.

**Figure 3:**
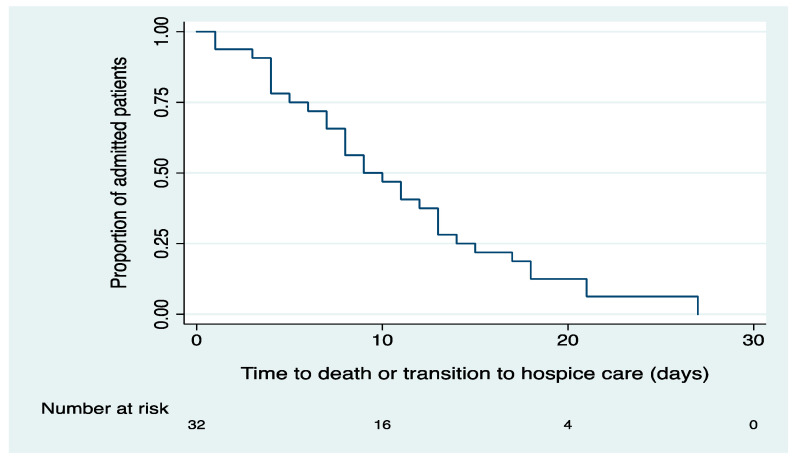
Time from admission to death/transition to hospice care in COVID-19 hospitalizations (n = 32; patients who died/transitioned to hospice).

For those who met the primary outcome, the mean length of stay (LOS) was 10.9 days (median: 9.5 days, SD: 6.9, range: 1–27 days). The median overall survival by the Kaplan–Meier analysis was nine days (Figure 3).

#### Impact of Socio-Demographic Factors on Primary Outcome in Hospitalized Patients

Using a multivariable logistic regression model, we analyzed odds of mortality/transition to hospice care for those hospitalized, after adjusting for age, gender, race, burden of comorbidities, and socio-economic status. Factors associated with increased odds of primary outcome included age >70 years versus ≤70 years and male (Table 3).

### 2.3. Secondary Outcomes

#### 2.3.1. Aggressive Inpatient Care during COVID-19-Related Hospitalization

Of those who were hospitalized, 41.1% (n = 30) were admitted to the ICU. Of these, 76.7% (n = 23) were intubated, 63.3% (n = 19) needed vasopressor support, and 23.3% (n = 7) needed emergent institution of renal replacement therapy. A comparison of baseline characteristics of those who had an ICU admission versus those managed on the general floor is shown in Table 4. A higher proportion of patients admitted to the ICU (66.7%, n = 20) recently received cancer-directed therapy versus those admitted to the general floor (39.5%, n = 17), and this difference was statistically significant (Chi-square *p*-value of 0.023).

#### 2.3.2. Length of Stay for Those Discharged after COVID-19-Related Hospitalization 

Amongst those discharged after a COVID-19-related hospitalization, the mean LOS was 7.6 days (median: 6.50 days, SD: 5.26, range: 1–24 days). The Kaplan–Meier analysis showed that 50% of the patients were discharged by day 6 and 89.5% by day 12. The mean LOS was significantly longer for those admitted to the ICU versus those admitted to the general floor (16.5 days vs 6.62 days, Mann–Whitney test *p*-value of 0.014).

## 3. Discussion

In our retrospective analysis, we analyzed the outcomes of patients with active invasive cancer and COVID-19 infection treated at a tertiary health care system in Detroit. Older age and male gender were found to be independent predictors of death/transition to hospice care amongst those hospitalized. On the other hand, race, burden of comorbidities, and median household income by zip code did not impact this outcome (Table 3). This could in part be related to an inadvertent selection bias. About two-thirds of the patient population had a CCI > 3, signifying the high burden of concomitant comorbidities in our patient population (Table 1). Moreover, our patient population was predominantly African American (65.5%) and most (63.3%) resided in zip codes with a median household income lower than that for the entire state of Michigan. This is consistent with the social demographics of Detroit, where 78.6% of the population is African American by ethnicity [14]. This could also be a plausible explanation as to why prostate cancer was the predominant primary site of malignancy in our study population versus lung cancer in the study from Wuhan [15]. Age and gender distribution in our study population were consistent with the data published earlier [8,10,16,17,18,19,20]. While chronic kidney disease and cardiac disease have been associated with poorer outcomes in those with COVID-19 infection [21,22], no difference was noted in terms of burden of comorbidities, based on primary outcome in our study (Table 1).

An overwhelming proportion of patients (85.5%) in our study ended up being hospitalized of which 41.1% were admitted to the ICU and 53.4% had a change of code status to DNR/DNI/CC. This was much higher than what was reported in the CCC19 study, where only 13.1% of patients with stable/progressing/responding cancers were admitted to the ICU [11]. A recently published study of hospitalized COVID-19 patients at the Henry Ford hospital reported an ICU admission in 24.2% and mortality in 18.1% of patients [12]. In our cohort of patients with active invasive cancer who were hospitalized with a COVID-19 infection, 43.8% died/transitioned to hospice. Of the 38 patients discharged following COVID-19 hospitalization, 23.7% were sent on home oxygen. The outcomes mentioned above highlight the dismal clinical outcomes of COVID-19 infection in this cohort of patients. It underscores the utmost importance of mitigation strategies such as social distancing and aggressive testing in our patients with active invasive cancer.

Another interesting observation in our study was the association of recent cancer-directed therapy with adverse clinical outcome. In our study population, more than half had received recent cancer-directed therapy which included systemic non-hormonal agents, hormonal agents, surgery, or radiation (Figure 2). Amongst those hospitalized, the proportion of patients who received recent cancer-directed treatment was no different based on the primary outcome (Table 1). However, a much higher proportion of those admitted to ICU (66.7%) had recently received cancer-directed treatment when compared to those admitted to general floors (39.5%). Furthermore, we observed a greater proportion (69.2% vs 37.5%, *p* = 0.08) of ICU admissions in those receiving non-hormonal systemic agents within 15 days preceding the diagnosis of COVID-19 infection, and this was in line with the findings of a Wuhan study [23]. ICU admissions were associated with a longer LOS of around 10 days when compared to general floor admissions. Moreover, the primary outcome was met in 85.2% of ICU admissions versus 20.9% of general floor admissions (Chi-square *p* < 0.001). The above findings are provocative and bring to question the safety of routine chemotherapy administration in patients with well-controlled, stable cancer when the risk of infection is high. The role of planned delays incorporated in cancer pathway algorithms for stable cancer patients on maintenance therapies needs to be evaluated in larger prospective studies. This is especially important given the potential of ongoing and future outbreaks of COVID-19 infection.

We noted higher levels of certain lab parameters for those who met the primary outcome versus those who did not, and this was consistent with previously reported studies [13,24]. Interestingly, severity of lymphopenia and ferritin elevation were not different between the two groups. Despite COVID-19 infection known to be associated with an elevated risk of thrombosis [25], only five patients were diagnosed with DVT/PE in our study and this probably reflects an under-diagnosis. Within our cohort of hospitalized patients, we did not notice any difference in terms of COVID-19-directed therapy received by those who died/transitioned to hospice versus those who did not. This is in contrast with the recently published study reporting better outcomes with Hydroxychloroquine [12]. A small sample size of our study is a limiting factor in terms of the ability to detect a difference. However, this observation is interesting, as well as thought-provoking and warrants further investigation.

There are many limitations to our study, primarily because of its retrospective nature. Data regarding the functional status of the patients were not included in the analysis. This could impact not only mortality but also the decision-making in terms of transition to hospice care or the change of code status. Differential treatment paradigms for COVID-19 infection were not controlled in our analysis. Finally, for those who were admitted, data from only the first COVID-related hospitalization were available and data from readmissions were not included.

## 4. Materials and Methods 

### 4.1. Study Design and Participants

**Figure 4:**
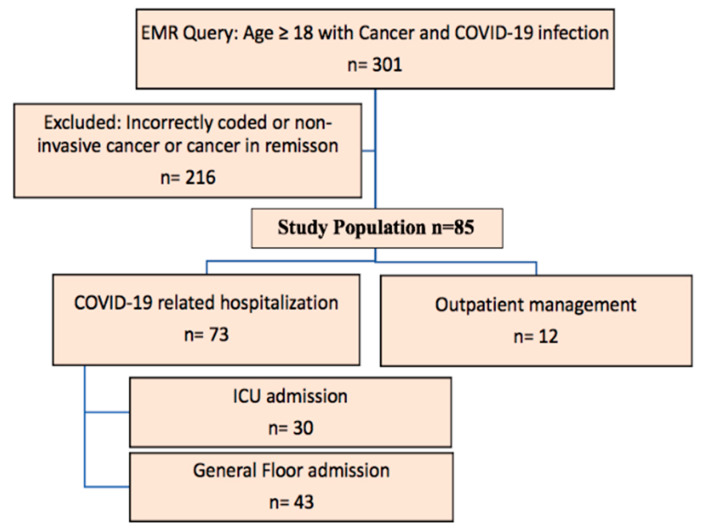
Study consort diagram.

This was a single institution retrospective study analyzing outcomes of patients with active invasive cancer who were treated for COVID-19 infection within the Henry Ford Health System, Detroit, Michigan. This research has been approved by the Henry Ford Health System institutional review board (ethic committee) on 20 April 2020 (ethic code: 13817) and requirement of informed consent was waived. Data were extracted from electronic medical records (EMR) using the International Classification of Diseases, Tenth Revision, Clinical Modification ICD-10 diagnosis codes to identify patients ≥18 years of age with cancer and COVID-19 infection (from 10 March 2020 to 17 April 2020). Evidence of severe acute respiratory syndrome coronavirus 2 (SARS-CoV-2) infection was defined by the documentation of positive results on polymerase chain reaction testing of a nasopharyngeal sample. A total of 301 patients were identified to have a diagnosis of cancer and COVID-19 infection. Active cancer was defined as hematological or solid malignancy which is either stable or progressing or responding. Patients could be under surveillance/observation or active treatment. Patients with no evidence of disease or cancer in remission or past history of cured cancer were not included. Furthermore, patients with premalignant conditions were not included. EMR of all 301 patients were reviewed and only patients with active and invasive cancer were included to arrive at our final study population of 85 patients. Clinical outcomes were monitored until 3 May 2020, the final date of follow-up. Figure 4 shows the consort diagram for the study.

### 4.2. Materials

Data included age, sex, self-identified race, and the zip code of the patient’s residence. To protect patient identity, an age over 89 was reported as 90. Burden of comorbidities was estimated using the Charlson Comorbidity Index score (CCI score). Median household income was estimated using an online tool (incomebyzipcode.com, accessed on 3 May 2020), based on current Census Bureau income statistics for US zip codes. Data from only the first COVID-19 infection-related hospitalization was included for those who were admitted during the study period. Data on aggressive medical interventions during hospital stay (includes intensive care unit stay, use of vasopressors, endotracheal intubation, and institution of emergent renal replacement therapy) were collected. Laboratory parameters previously identified as being associated with adverse risk were also recorded [5,9,13,24,26]. These included peak values of serum creatinine (Cr), aspartate aminotransferase (AST), alanine aminotransferase (ALT), Lactate dehydrogenase (LDH), creatine phosphokinase (CPK), D-Dimer, markers of inflammation [Ferritin, C-reactive protein (CRP), interleukin-6 (IL-6), and Triglyceride], and markers of cardiac injury [High sensitivity troponin (Hs-Trop) and brain natriuretic peptide (BNP)]. Nadir values of lymphocyte count, absolute neutrophil count (ANC), and platelet count were also collected. The nadir value of ANC was collected for its association with bone marrow suppression amongst those receiving chemotherapy [27]. Moreover, nadir values platelets were collected because the COVID-19 infection has been associated with thrombocytopenia [28]. Of note, patients with chronic kidney disease/end stage renal disease were excluded from the analysis of serum Cr. Furthermore, patients with congestive heart failure were excluded from the analysis of Hs-Trop and BNP. Data about COVID-19-directed treatment were collected. The site of primary malignancy and presence of metastases in those with solid cancer was noted. Moreover, cancer-directed treatment administered within the 3 months preceding a COVID-19 infection (“recent cancer treatment”) was recorded. Mortality, transition to hospice care, change in the code status to do-not-resuscitate or do-not-intubate or comfort care (DNR/DNI/CC), and documentation by imaging of deep vein thrombosis or pulmonary embolism (DVT/PE) following COVID-19 infection was collected. Length of stay (LOS) and need of home oxygen at discharge were some other variables recorded specifically for those hospitalized.

### 4.3. Statistical Analysis

Continuous variables were summarized using mean and standard deviation (SD) or median and range. Categorical variables were summarized as the counts and percentages in each category. A two-sample Wilcoxon rank-sum (Mann–Whitney) test was applied to continuous variables. Pearson’s Chi-square/Fisher’s exact tests were used for categorical variables as appropriate. The primary end point of the study was mortality/transition to hospice care following COVID-19 infection and was analyzed for the hospitalized patients. Secondary outcomes were only measured for the hospitalized patients and included LOS and the need of aggressive medical interventions. These outcomes were reported using descriptive statistics. Logistic regression was used to model the effect of independent variables on the attainment of primary outcome. Overall survival (OS), defined as time from admission to death or transition to hospice care, was analyzed for those hospitalized using the Kaplan–Meier method. A *p*-value of 0.05 or less was considered to be statistically significant. Statistical analysis was performed using the STATA statistical software (Stata/IC 15.1).

## 5. Conclusions

Our findings highlight high rates of death or transition to hospice and utilization of aggressive inpatient care following COVID-19 infection in our predominantly inner-city patient population with active invasive cancer. The findings of our study quantify the health care disparity this ongoing pandemic has uncovered. Men and those older than 70 years of age had a greater than four-fold increase in odds of dying or being transitioned to hospice care when compared to females and those ≤70 years of age. In addition to this, we noted that a significantly higher proportion of patients admitted to the ICU had received cancer-directed treatment within the preceding three months. Collectively, these findings underscore the need for aggressive measures for preventing COVID-19 exposure in those with active invasive cancer and the urgent need to bridge gaps in health care access. Prospective trials to evaluate the role for continued chemotherapy versus planned treatment breaks for stable patients on maintenance chemotherapy also need to be conducted.

## Figures and Tables

**Table 1:** Baseline characteristics.

Factors	All Patients n = 85 (%)	Hospitalized Patients n = 73 (%)		
		Death/Transition to Hospice Documented n = 32	Death/Transition to Hospice not Documented n = 41	Chi-square *p*-Value
**Males**	47 (55.3)	25 (78.1)	18 (43.9)	0.003
**Age ≤70 Years**	50 (58.5)	12 (37.5)	27 (65.8)	0.016
**African Americans**	55 (65.5)	20 (62.5)	27 (67.5)	0.462
**Local household income below the state median**	50 (63.3)	18 (56.2)	25 (60.9)	0.684
**Charlson Comorbidity Index Quartiles (CCI score range)**	*n = 76*	*n = 31*	*n = 37*	0.081
Quartile 1 (1–3)	25 (32.8)	7 (22.5)	13 (35.1)	
Quartile 2 (4–6)	23 (30.2)	14 (45.1)	7 (18.9)	
Quartile 3 (7–8)	15 (19.7)	7 (22.5)	8 (21.6)	
Quartile 4 (9–16)	13 (17.1)	3 (9.6)	9 (24.3)	
**Cancer Type**				0.351
Metastatic Solid	31 (36.5)	12 (37.5)	14 (34.1)	
Non-metastatic Solid	32 (37.6)	10 (31.2)	19 (46.3)	
Hematological	22 (25.9)	10 (31.2)	8 (19.5)	
**COVID-19-related treatment**				
Steroids	51 (60)	24 (75.0)	27 (65.8)	0.398
Hydroxychloroquine	62 (72.9)	27 (84.4)	34 (82.9)	0.868
Azithromycin	21 (24.7)	8 (25.0)	11 (26.8)	0.860
Tocilizumab	3 (3.5)	2 (6.2)	1 (2.4)	0.416
**Recent Cancer-directed treatment**	47 (55.3)	17 (53.1)	20 (48.8)	0.713
Hormonal agents	11 (12.9)			
Non-Hormonal systemic agents:				
Cytotoxic agents	19 (22.3)			
Immunotherapy	6 (7.1)			
Others	15 (17.6)			
Radiation therapy	6 (7.1)			
Surgery	4 (4.7)			
**ICU admission**	30 (35.3)	23 (71.9)	7 (17.1)	<0.001
**Coded status updated to DNR/DNI/CC**	39 (45.9)	30 (93.7)	9 (21.9)	<0.001

**CCI:** Charlson Comorbidity Index, **ICU:** intensive care unit, **DNR/DNI/CC:** do-not-resuscitate/do-not-intubate/comfort care.

**Table 2:** Lab parameters.

Lab Parameters (Mean Values)	All Patients (n = 85)	Hospitalized Patients (n = 73)		
	Overall Mean ± SD, Median [min, max]	Death/Transition to Hospice Documented Mean ± SD (n = 32)	Death/Transition to Hospice Not Documented Mean ± SD (n = 41)	Two-Sample Wilcoxon Rank-Sum (Mann–Whitney) Test *p*-Value
***Nadir Values***				
**Lymphocyte count (k/µL)**	1.1 ± 3.1	1.6 ± 4.7 (32)	0.7 ± 1.2 (41)	0.906
	0.5 [0, 24.8]			
**ANC (k/µL)**	3.9 ± 3.2	5.1 ± 4.1 (32)	3.1 ± 2.1 (41)	0.014
	3.1 [0.2, 20.4]			
**Platelet count (k/µL)**	163.8 ± 116.3	146.7 ± 96.8 (32)	178.8 ± 133.4 (41)	0.256
	149 [24, 900]			
***Peak Values***				
**Serum Cr (mg/dL)**	1.8 ± 1.9	2.4 ± 2.3 (19)	1.5 ± 1.5 (32)	0.019
	1.2 [0.6, 7.9]			
**AST (IU/L)**	104.8 ± 154.4	147.1 ± 206.9 (30)	78.4 ± 98.2 (40)	0.013
	56 [14, 1031]			
**ALT (IU/L)**	66.5 ± 102.6	86.1 ± 142.1 (30)	55.4 ± 61.6 (40)	0.264
	31 [6, 775]			
**LDH (IU/L)**	513.1 ± 332.2	616.7 ± 375.7 (30)	437.1 ± 277.2 (41)	0.007
	405 [112, 1812]			
**CPK (IU/L)**	425.3 ± 812.2	586.2 ± 1054.1 (29)	311.6 ± 572.9 (41)	0.079
	146 [18, 5270]			
**Triglyceride (mg/dL)**	216.3 ± 126.4	234.9 ± 145.6 (22)	190.8 ± 92.5 (16)	0.604
	175.5 [47, 602]			
**Hs-Troponin (ng/L)**	855.1 ± 3414.9	1316.4 ± 4417 (20)	195.9 ± 542.2 (14)	0.004
	101 [19, 20,000]			
**BNP (pg/mL)**	230.7 ± 378.5	399.3 ± 511.1 (18)	98.8 ± 131.6 (23)	0.004
	75 [9, 1744]			
**Ferritin (ng/mL)**	3470.6 ± 10030.3	5839.3 ± 14863.3 (31)	1679.6 ± 2291.7 (41)	0.084
	1277.5 [52, 78,689]			
**CRP (mg/dL)**	17.4 ± 10.5	21.2 ± 11.5 (28)	14.8 ± 9.0 (41)	0.019
	15.3 [0.1, 46.1]			
**IL-6 (pg/mL)**	126.4 ± 185.4	163.3 ± 209.2 (17)	36.7 ± 42.2 (7)	0.092
	39.5 [5, 582]			
**D-Dimer (µg/mL)**	5.5 ± 6.8	8.1 ± 9.1 (27)	3.6 ± 3.7 (39)	0.011

**ANC:** absolute neutrophil count, **Serum Cr**: serum creatinine, **AST**: aspartate aminotransferase, **ALT:** alanine aminotransferase, **LDH:** Lactate dehydrogenase, **CPK:** creatine phosphokinase, **CRP**: C-reactive protein, **IL-6:** interleukin-6, **Hs-Trop:** high sensitivity troponin, **BNP:** brain natriuretic peptide.

**Table 3:** Multivariate analysis: factors associated with primary outcome in hospitalized patients.

Factor	Odds Ratio for Mortality/Transition to Hospice	*p*-Value	95% Confidence Interval
**Age: >70 vs. ≤70 Years**	4.7	0.012	1.3–15.9
**Gender: Male vs. Female**	4.8	0.008	1.5–15.8
**Race: African American vs. Caucasian**	2.2	0.227	0.6–8.3
**Charlson Comorbidity Index score**	0.9	0.793	0.8–1.1
**Median household income for zip code of residence ($): ≥54,938 vs. <54,938**	0.8	0.769	0.2–2.7

**Table 4:** Baseline characteristics of hospitalized patients (n = 73) based on admission to the intensive care unit (ICU).

Factors	Admitted to ICU n = 30 (%)	Admitted to General Floor n = 43 (%)	Chi-Square *p*-Value
Males	20 (66.7)	23 (53.5)	0.260
Age ≤70 Years	16 (53.3)	23 (53.5)	0.990
African Americans	21 (72.4)	26 (63.4)	0.430
Died/Transitioned to Hospice care	23 (85.2)	9 (20.9)	<0.001
Local Household Income Below the State Median	17 (56.6)	26 (60.5)	0.756
Cancer Type			0.896
Metastatic Solid	11 (36.7)	15 (34.9)	
Non-metastatic Solid	11 (36.7)	18 (41.9)	
Hematological	8 (26.6)	10 (23.2)	
Received Steroids	23 (76.7)	28 (65.1)	0.290
Received Hydroxychloroquine	28 (93.3)	33 (76.7)	0.060
Received Azithromycin	9 (30)	10 (23.3)	0.518
Received Tocilizumab	3 (10)	0 (0)	0.065
Recent Cancer-directed Therapy	20 (66.7)	17 (39.5)	0.023

## Supplementary Materials

The following are available online at https://www.mdpi.com/2072-6694/12/9/2377/s1, Table S1: List of Abbreviations used in Figure 2.
