# Supplementary material for: COVID-19 and Cancer: Lessons Learnt from a Michigan Hotspot
Source: Cancers (Basel). 2020 Aug 22;12(9):2377. doi: 10.3390/cancers12092377 (PMC7565165; doi:10.3390/cancers12092377)
Supplement: Supplementary file 1 [file cancers-12-02377-s001.zip › cancers-906758-supplementary.docx]

Supplementary Table

COVID-19 and Cancer: Lessons Learnt from a Michigan Hotspot

Sunny R. K. Singh, Kannan Thanikachalam, Hiba Jabbour-Aida, Laila M. Poisson and Gazala Khan

**Table S1.** List of Abbreviations used in Figure 2.

| **Primary malignancy** | |
| --- | --- |
| CLL | Chronic lymphocytic leukemia |
| CML | Chronic myelogenous leukemia |
| AML | Acute myeloid leukemia |
| **Systemic Agent** | |
| CyBorD | Cyclophosphamide, Bortezomib and Dexamethasone |
| VRd | Bortezomib, Lenalidomide and Dexamethasone |
| 5-FU | 5-Fluorouracil |
| R-GemOx | Rituximab, Gemcitabine and Oxaliplatin |
| mFOLFOX6 | (Modified) 5-Fluorouracil, leucovorin and Oxaliplatin |
| DRd | Daratumumab, Lenalidomide and Dexamethasone |
